# Desmoplastic small round cell tumor: a case report and review of the literature

**DOI:** 10.1186/1477-7819-12-9

**Published:** 2014-01-10

**Authors:** Xiang Li, Jing Yu, Shibao Fang, Xiaoming Xing, Jie Zhao

**Affiliations:** 1 Department of Ultrasound, Affiliated Hospital of Medical College Qingdao University, Qingdao 266003, China; 2 Department of Pathology, Affiliated Hospital of Medical College Qingdao University, 16 Jiangsu Road, Qingdao 266003, China

**Keywords:** Desmoplastic small round cell tumor, Imaging, Pathology

## Abstract

Desmoplastic small round cell tumor is a rare malignant tumor that has a poor prognosis. It affects predominantly young males. In the current report, a 14-year-old male patient was admitted to the hospital for evaluation of abdominal distension, and abdominal pain. Imaging examination revealed a high prevalence of multiple intraperitoneal and liver parenchymal cystic and solid tumors. After an explorative surgery, the pathological findings confirmed the presentation of desmoplastic small round cell tumor. Diagnosis of desmoplastic small round cell tumor could easily have been overlooked since there was no specific evidence for this condition available in the clinical and imaging examinations. In the present study, ultrasound examination detected solid cystic masses, which suggested the presence of necrosis and hemorrhage. Immunohistochemistry and cytogenetic studies confirmed the diagnosis of desmoplastic small round cell tumor in this patient.

## Background

Desmoplastic small round cell tumor (DSRCT) is a highly malignant small round cell tumor. It was first described by Gerald and Rosai in 1989 and formally named in 1991 [1,2]. It is frequently prevalent in the abdominal and pelvic cavities of adolescent males with a potential for multidirectional differentiation. The mechanism of histogenesis of this tumor remains unclear but might be derived from multipotential differentiated primitive mesenchymal cells or neuroectodermal and primitive mesenchymal tissue. The ethics committee of the Affiliated Hospital of Qingdao University Medical College, China approved this case study. Informed consent was signed by the recruited patient.

## Case presentation

**Figure 1:**
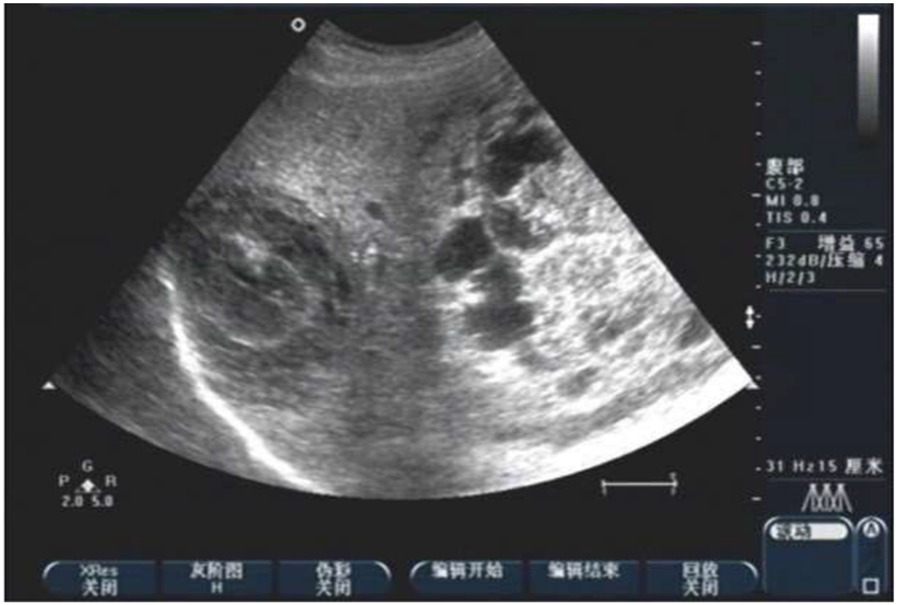
Ultrasound images of the mass in the right liver and abdominal cavity.

**Figure 2:**
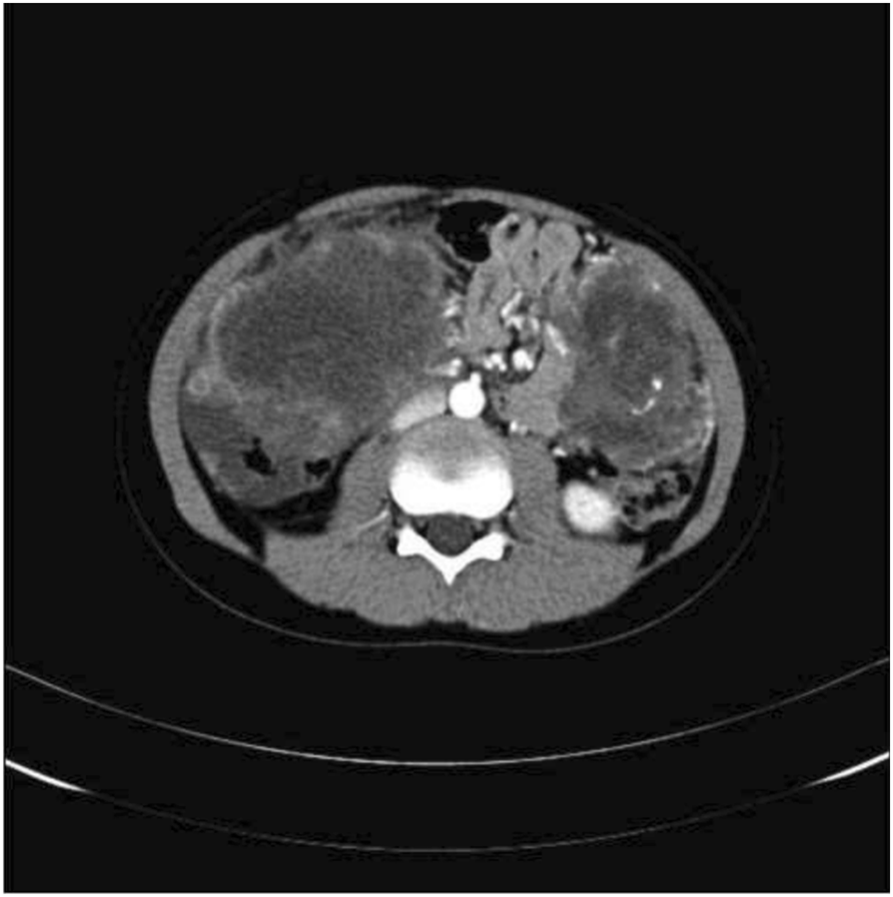
Enhanced CT image in the arterial phase of the mass in the right liver and abdominal cavity.

A 14-year-old male patient was admitted to our hospital for evaluation of abdominal distension, and abdominal pain. A physical examination revealed an apparent abdominal mass and tenderness. The mass exhibited an unclear boundary and hard texture. A low degree of mobility was also observed. Ultrasonography (US) of the abdomen revealed multiple cystic-solid masses with regular shape and clear boundaries in the right liver and abdominal cavity with diameters of 12.1 cm to 4.9 cm. In addition, there was a mass in the hilum of the spleen (Figure 1). These findings were confirmed by enhanced computed tomography (CT) of the abdomen (Figure 2).

The DSRCT cell differentiation direction is uncertain, but this tumor can express epithelial, mesenchymal, neuroendocrine, and other immunophenotypes and is cytogenetically specific. In addition, cells can generate the EWS-WT1 fusion gene. DSRCT is a very rare soft tissue tumor.

**Figure 3:**
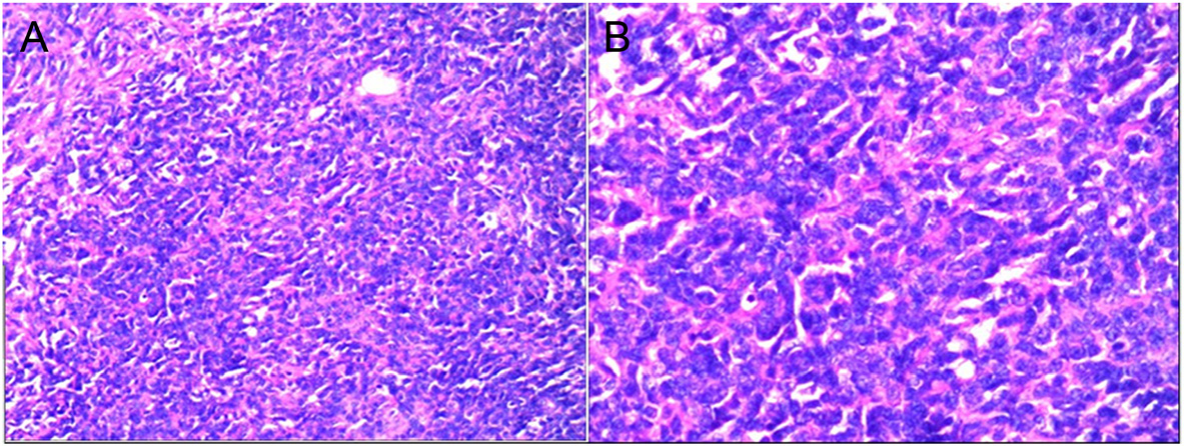
**Histological findings of desmoplastic small round cell tumor. A**. HE 10×; **B**. HE 20×.

**Figure 4:**
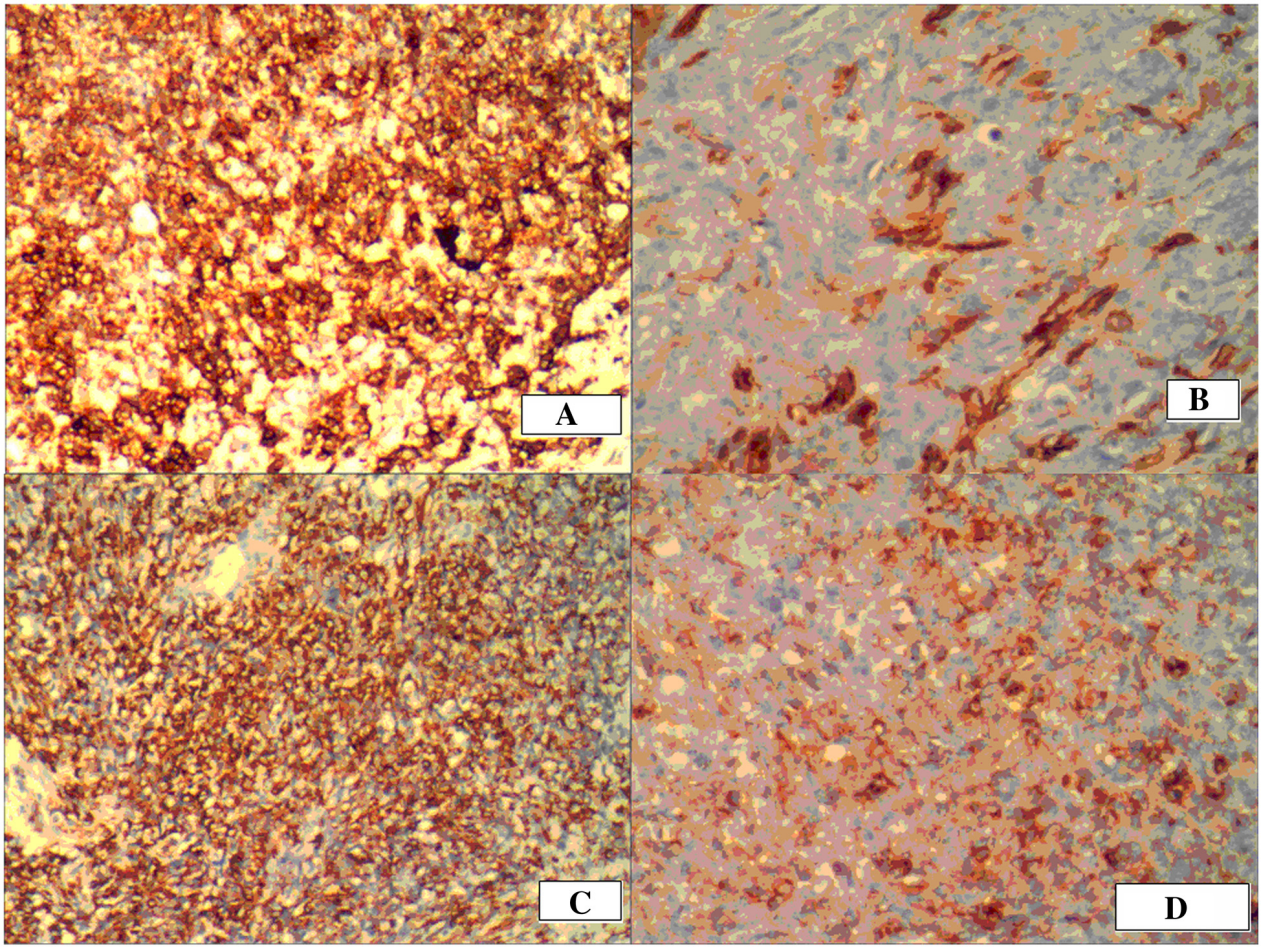
**Immunohistochemistry of desmoplastic small round cell tumor. A**. AE1/AE3; **B**. EMA; **C**. Desmin; **D**. Vimtin.

During the operation, liver segments 5 and 6 that had involved tumor were removed. The portion of the tumor located in hepatic segment 7 was also resected. The hepatic wound was repaired. A mass with a hard texture and nodular surface in the left abdominal cavity was excised. The mass observed in the hilum of the spleen presented with both the splenic artery and vein tightly adhered to the tumor. For this reason, they were completely excised. The pathological findings of the tumor in the abdominal cavity and right hepatic lobe confirmed that the tumor was a multifocal malignant DSRCT. There were multifocal metastatic nodules within the greater omentum. Immunohistochemistry gave the following phenotypic markers: desmin (++), CK (+), Vim (+/-), epithelial membrane antigen.

(EMA) (+/-), CD34 (-), CD117 (-), neuron-specific enolase (NSE) (-), WT-1 (-), and S-100 (-) Figures 3 and 4.

After the operation, it was planned he should receive seven courses of chemotherapy. Up to date, he has already undergone all cycles of chemotherapy and he is in good general condition.

## Discussion

DSRCT has the same characteristics as those of other small round cell tumors, such as Ewing's sarcoma, small cell mesothelioma, neuroblastoma, and lymphoma [3]. Thus, it is also classified as an undifferentiated soft tissue tumor [4-7]. Histologically, DSRCT cells are closely arranged and can form epithelioid cells of varying sizes and shapes. A large involvement of fibrous connective tissue is observed around and among tumor cell nests [3].

In most cases of DSRCT desmin, CK EMA,and vimentin are positive, of which desmin and CK being positive at the same time is considered DSRCT’s most specific immunological indexes. Stromal elements are vimentin positive, prompt from the muscle fiber mother cell.The direction of DSRCT cellular differentiation is largely uncertain. However, this tumor can express epithelial, mesenchymal, neuroendocrine, and other immunophenotypes and is cytogenetically specific. In addition, cells can generate the EWS-WT1 fusion gene [8].

Since it may metastasize to the liver, most commonly, and lungs, and or lymph nodes of the groin,neck, or mediastinum, once abdominal imaging reveals multiple tumors, imaging of the chest with a CT scan and distant metastasis using a total body positron emission tomography (PET) scan, is recommended as the preferred. Percutaneous or open biopsy of the lesion should be evaluated by immunohistochemistry and cytogenetics to confirm the characteristics of DSRCT.

DSRCT mainly occurs in children and adolescents. Primary peritoneal cases account for the vast majority of appearances of this disease, and a few cases are reported in single organs, such as the ovary, lung, kidney, pancreas, bone, and posterior cranial fossa [9-11]. Clinical manifestations include abdominal distension, abdominal discomfort, abdominal pain, and an abdominal mass accompanied by constipation, dysuria, umbilical hernia, intestinal obstruction, and other compressive symptoms.

Some patients may suffer from acute abdominal discomfort or cannot walk upright because of abdominal pain. The majority of patients develop a reduced body weight compared with that before the onset. Physical examination reveals an apparent mass that is palpable in the abdominal cavity that is accompanied by tenderness, an unclear mass boundary, hard texture, and a low degree of motility. Ultrasound, CT scanning, and magnetic resonance imaging (MRI) often display a large nodular mass within the pelvic or abdominal cavity, mostly in the greater omentum or mesentery, while no apparent primary lesion is detected in the parenchymal organs. In most cases, the lesions are multifocal, and some patients might present with single nodular lesions. In some cases, paravertebral involvement is also visible. Even the peritoneum may be thickened in a diffuse and nodular manner, similar to that of mesothelioma. In other cases, liver metastases occur, accompanied by ascites. Diagnosis only through noninvasive imaging examinations is difficult.

In the present study, ultrasound examination detected cystic-solid masses, suggesting the occurrence of necrosis and hemorrhage. Abdominal ultrasonography findings were also highly suspicious and suggested a multifocal cystic-solid mass in the abdominal or pelvic cavity and other large organs consistent for the presentation of DSRCT. A definitive diagnosis is only obtained based on pathological findings.

Current therapies are mainly dependent on surgical resection, chemotherapy, and radiotherapy. According to the patient’s condition, interventional therapy can be given, and preoperatively induced chemotherapy, surgery, and radiotherapy. Recent studies show that complete surgical excision, including a 1 to 2 mm- long implant, is necessary to achieve long-term disease control. Intraperitoneal hyperthermic chemotherapy using cisplatin is thought to be effective at reducing the recurrence rate of disease [12]. Nevertheless, patients presenting with DSRCT have a poor prognosis, with only a 29% actuarial three-year survival rate and an 18% five-year survival rate [13,14].

## Conclusion

DSRCT is a rare malignant tumor with a poor prognosis, and it mainly affects young males. Imaging examination alone is not sufficient for a definitive diagnosis, and immunohistochemistry and cytogenetic studies are suggested.

## Competing interests

The authors declare that we have no competing interests.

## Authors’ contribution

JZ, and XX, carried out the pathology, JY, follow up cases, XL, and SF, participated in drafted the manuscript. All authors read and approved the final manuscript.
